# Handling underlying discrete variables with bivariate mixed hidden Markov models in NONMEM

**DOI:** 10.1007/s10928-019-09658-z

**Published:** 2019-10-26

**Authors:** A. Brekkan, S. Jönsson, M. O. Karlsson, E. L. Plan

**Affiliations:** Department of Pharmaceutical Biosciences, Uppsala University, Box 591, 75124 Uppsala, Sweden

**Keywords:** Hidden Markov model, HMM, Mixed effects, Parameter estimation, NONMEM

## Abstract

Non-linear mixed effects models typically deal with stochasticity in observed processes but models accounting for only observed processes may not be the most appropriate for all data. Hidden Markov models (HMMs) characterize the relationship between observed and hidden variables where the hidden variables can represent an underlying and unmeasurable disease status for example. Adding stochasticity to HMMs results in mixed HMMs (MHMMs) which potentially allow for the characterization of variability in unobservable processes. Further, HMMs can be extended to include more than one observation source and are then multivariate HMMs. In this work MHMMs were developed and applied in a chronic obstructive pulmonary disease example. The two hidden states included in the model were remission and exacerbation and two observation sources were considered, patient reported outcomes (PROs) and forced expiratory volume (FEV1). Estimation properties in the software NONMEM of model parameters were investigated with and without random and covariate effect parameters. The influence of including random and covariate effects of varying magnitudes on the parameters in the model was quantified and a power analysis was performed to compare the power of a single bivariate MHMM with two separate univariate MHMMs. A bivariate MHMM was developed for simulating and analysing hypothetical COPD data consisting of PRO and FEV1 measurements collected every week for 60 weeks. Parameter precision was high for all parameters with the exception of the variance of the transition rate dictating the transition from remission to exacerbation (relative root mean squared error [RRMSE] > 150%). Parameter precision was better with higher magnitudes of the transition probability parameters. A drug effect was included on the transition rate probability and the precision of the drug effect parameter improved with increasing magnitude of the parameter. The power to detect the drug effect was improved by utilizing a bivariate MHMM model over the univariate MHMM models where the number of subject required for 80% power was 25 with the bivariate MHMM model versus 63 in the univariate MHMM FEV1 model and > 100 in the univariate MHMM PRO model. The results advocates for the use of bivariate MHMM models when implementation is possible.

**Electronic supplementary material:**

The online version of this article (10.1007/s10928-019-09658-z) contains supplementary material, which is available to authorized users.

## Introduction

Non-linear mixed effects models (NLMEs) are typically restrained to handle stochastic processes in observed variables; in contrast, hidden Markov models (HMMs) are a class of statistical models that can be used to characterize relationships between observed variables and unobserved stochastic processes. HMMs can, based on recorded data, shed light on unobservable (hidden) processes, categorized as states, such as the theoretical notion of disease status. Describing unobserved variables may be of importance to describe the system of interest and make inferences, for instance about drug effects influencing an underlying disease status that cannot be observed directly. Ignoring such influences may cause bias in estimates [1]. Further, disease progression modelling is often of interest, which may not be possible if the actual disease status is unobservable. In such cases, HMMs may be used to obtain the most likely underlying state sequences, a representation of the disease status sequence. The attractive properties of such latent (hidden) variable models, including their flexibility and, often, higher power to detect a covariate or drug effect, have been described in multiple instances [1–3].

HMMs handle unobserved processes through a time series chain, hypothesizing multiple hidden states [4, 5]. Typically set up as a discrete time chain, the series of states visited takes place among a defined set of possible states (often two of them, but nothing precludes a higher number). The key estimated parameters are therefore transition probabilities between the states, like in a classical Markov model [4], relaxing the assumption of observations being independent. In a first-order Markov chain, the transition probabilities give the probability of transitioning to a certain state, given the previous state. Mixed hidden Markov models (MHMMs) extend HMMs to population data, allowing for the estimation of random effect parameters and potentially, covariate effects [6–8]. These models present greater flexibility since random effects can be incorporated on parameters associated with either observations, or hidden states.

Most HMMs presented in the literature deal with one observed variable which tends to be of a count nature (often described with a Poisson-like distribution). Examples include lesion counts (observed variable), revealing whether multiple sclerosis patients are in a relapse or remission (hidden states) [6], the number of seizures (observed variable), sorting days between low and high epileptic activity (hidden states) [8], or CD4 counts (observed variable), affected whether HIV positive patients present an unknown concomitant infection or not (hidden states) [1]. However measures are only partial representations of the truth; hence, multiplying the number of measures taken into account and analyzing them together should allow for a more precise and less biased assessment of an underlying disease status. For these reasons, in this work, we were interested in exploring how HMMs can be extended to include multiple sources of observations (multivariate HMMs). More precisely, the objective of this work was to develop a bivariate MHMM that depend on two, potentially correlated, simultaneously observed, continuous variables.

The application example chosen for this work is chronic obstructive pulmonary disease (COPD), a condition that afflicts approximately 65 million people worldwide [9] and is predicted to increase over the coming years with the rising age of the world’s population. With intermittent periods of no deterioration in lung function (remission) and periods where lung function is compromised (exacerbations) [10], the diagnosis and management of COPD is difficult as well as the investigation of treatment effects on the disease progression [11, 12]. The severity of the symptoms can be measured with endpoints such as the forced expiratory volume in one second (FEV1) or patient recorded outcomes (PROs). Incorporating the observed variables, FEV1 and PRO, simultaneously in the analysis of COPD data may give insight on the hidden patient disease status, i.e. whether patients are in remission or whether they are experiencing an exacerbation.

The present work aims at presenting a new type of multivariate MHMM, exploring its implementation in the software NONMEM, and investigating its benefits in drug development, through a series of simulation-estimation analyses. Specific questions were: (i) how do random effect- and covariate (including drug effect) relationship magnitudes affect parameter estimation accuracy and precision, (ii) how well is the correlation between the two observed variables estimated with the bivariate model and what is the impact of ignoring it, and (iii) what is the power to detect a drug effect with a bivariate MHMM incorporating both observation sources simultaneously compared to two separate univariate MHMMs?

## Methods

### Models

A bivariate MHMM was developed, combining FEV1 measurements and PROs. The model included two hidden Markov states, representing COPD disease in remission (R) or during exacerbation (E). The initial parameter values (Table 1) for the model were chosen based on clinical plausibility.

**Table 1:** Reference parameter values used in the bivariate mixed hidden-Markov model

Parameter (unit)	Value	Description
Observed variable parameters		
$\theta_{{{\text{FEV}}1_{\text{R}} }} \left( {{\text{L}}/{\text{s}}} \right)$	2.00	The mode of the distribution of FEV1 in remission
$\theta_{{{\text{FEV}}1_{\text{E}} }} \left( {{\text{L}}/{\text{s}}} \right)$	0.25	The mode of the distribution to be subtracted from FEV1_R_ in the exacerbation state
$\theta_{{{\text{PRO}}_{\text{R}} }} \left( {\text{score}} \right)$	2.50	The mode of the distribution of PRO in remission
$\theta_{{{\text{PRO}}_{\text{E}} }} \left( {\text{score}} \right)$	0.5	The mode of the distribution to be added to PRO_R_ in the exacerbation state
Hidden state parameters		
INIT	0.90	Initial state probability of being in remission
$\theta_{{\pi_{RE} }}$	0.05	Transition probability from remission to the exacerbation state
$\theta_{{\pi_{ER} }}$	0.15	Transition probability from the exacerbation state to remission
SLP	1.00	Hypothetical drug effect reducing the probability of transitioning from remission to the exacerbation state
Variance parameters		
$\omega_{{FEV1_{R} }}^{2}$	0.03	Interindividual variability of the mode of FEV1 in remission
$\omega_{{FEV1_{E} }}^{2}$	0.03	Interindividual variability of the mode of FEV1 in the exacerbation state
$\omega_{{PRO_{R} }}^{2}$	0.09	Interindividual variability of the mode of PRO in remission
$\omega_{{PRO_{E} }}^{2}$	0.09	Interindividual variability of the mode of PRO in the exacerbation state
$\omega_{{\pi_{RE} }}^{2}$	0.06	Interindividual variability of $\pi_{RE}$
$\sigma_{FEV1}^{2}$	0.015	The variance (residual error) of the distribution of FEV1 in both states
$\sigma_{PRO}^{2}$	0.05	The variance (residual error) of the distribution of PRO in both states
$\rho_{R}$ and $\rho_{E}$	− 0.33	The correlation between the two variables

Both the FEV1 and PRO model components were composed of two continuous functions, depending on whether the observation was made while the patient status was remission or exacerbation. The functions included fixed effects parameters as well as random effects parameters accounting for inter individual variability (IIV). Individual FEV1 values were modelled assuming log normal distributions:

1 $$FEV1_{R} = \theta_{{FEV1_{R} }} \cdot \exp \left( {\eta_{{FEV1_{R} }} } \right),$$

2 $$FEV1_{E} = FEV1_{R} - \left( {\theta_{{FEV1_{E} }} \cdot \exp \left( {\eta_{{FEV1_{E} }} } \right)} \right),$$

where $FEV1_{R}$ and $FEV1_{E}$ are the individual values of FEV1 in remission and exacerbation, respectively, $\theta_{{FEV1_{R} }}$ and $\theta_{{FEV1_{E} }}$ are the population estimates of the mode of FEV1 in each state. $\eta_{{FEV1_{R} }}$ and $\eta_{{FEV1_{E} }}$ are random effects describing the deviation between individual and typical values, and are assumed to be normally distributed with a mean of 0 and variances of $\omega_{{FEV1_{R} }}^{2}$ and $\omega_{{FEV1_{E} }}^{2}$, respectively.

PRO scores were described with a time dependent decrease to represent a placebo effect (PE):

3 $$PRO_{R} = \left( {\theta_{{PRO_{R} }} + \eta_{{PRO_{R} }} } \right) \cdot \left( {1 - PE \cdot \left( {1 - \exp \left( {\frac{ - \log \left( 2 \right)}{PHL \cdot Time}} \right)} \right)} \right),$$

4 $$PRO_{E} = PRO_{R} + \left( {\theta_{{PRO_{E} }} + \eta_{{PRO_{E} }} } \right),$$

where log is the natural logarithm, $PRO_{R}$ and $PRO_{E}$ are the individual values of PRO in remission and exacerbation, respectively, $\theta_{{PRO_{R} }}$ and $\theta_{{PRO_{E} }}$ are the population estimates of the mode of PRO in each state. $\eta_{{PRO_{R} }}$ and $\eta_{{PRO_{E} }}$ are random effects describing the deviation between individual and typical values, and are assumed to be normally distributed with a mean of 0 and variances of $\omega_{{PRO_{R} }}^{2}$ and $\omega_{{PRO_{E} }}^{2}$, respectively. PHL is the placebo effect half-life.

In this model, the maximum decrease of PRO from baseline over time, i.e. PE, was simulated to be 20% (PE = 0.2), and was assumed to occur at approximately 50 weeks, i.e. with a PHL of 10 weeks. Patients suffering from COPD have a FEV1 that ranges from 0.5 to 2 L/s, and thus the modes of the distribution of FEV1 in remission and during exacerbation were set to 2 and 1.75 (Table 1), respectively. The modes of the PRO distributions in remission and exacerbation were set to 2.5 and 3 (Table 1), respectively, attempting to mimic the relative changes in score during exacerbation seen when using the Asthma control questionnaire (ACQ) [13].

The general description of an HMM is:

5 $$p\left( {Y_{1} , \ldots Y_{n} , Z_{1} , \ldots Z_{n} } \right) = p\left( {Z_{1} } \right)p\left( {Y_{1} |Z_{1} } \right)\mathop \prod \limits_{t = 2}^{n} p\left( {Z_{t} |Z_{t - 1} } \right)p(Y_{t} |Z_{t} )$$

where *Y*_*n*_ denotes an observation, *Z*_*n*_ is the hidden state, *t* is the time point, *n* is the number of observed time points, *p*(*Z*_*1*_) is the initial state probability, *p*(*Y*_*1*_|*Z*_*1*_) is the emission probability at the start of the time sequence (i.e. the probability of an observation given a certain hidden state), *p*(*Z*_*t*_|*Z*_*t*−*1*_) is the transition probability and *p*(*Y*_*t*_|*Z*_*t*_) is the emission probability at time *t* given state *Z* at time *t*.

The emission probabilities for FEV1 and PRO (Eq. 6), governing the distribution of the observed variables at a particular time given the state of the hidden variable at that time, were modeled incorporating additive residual error terms on each observed variable. Two emission probability functions were necessary since there were two states present in the model. These functions are part of the likelihood of the observed variables and were described by the normal probability density function (PDF), assuming that the variables were normally distributed:

6 $$P\left( {Y_{FEV1} ,Y_{PRO} |S = s} \right) = \frac{1}{{2\pi \sqrt {\sigma_{{FEV1_{s} }}^{2} \sigma_{{PRO_{s} }}^{2} \left( {1 - \rho_{s}^{2} } \right)} }} \cdot e^{{ - \frac{1}{{2(1 - \rho_{s}^{2} )}}\left( {\left( {\frac{{Y_{FEV1} - FEV1_{s} }}{{\sigma_{{FEV1_{s} }} }}} \right)^{2} - 2\rho_{s} \left( {\frac{{Y_{FEV1} - FEV1_{s} }}{{\sigma_{{FEV1_{s} }} }}} \right)\left( {\frac{{Y_{PRO} - PRO_{s} }}{{\sigma_{{PRO_{s} }} }}} \right) + \left( {\frac{{Y_{PRO} - PRO_{s} }}{{\sigma_{{PRO_{s} }} }}} \right)^{2} } \right)}} ,$$

where $Y_{FEV1}$ and $Y_{PRO}$ are the observed variables of interest, S is the state that can be either R or E for remission or exacerbation, respectively, *FEV1* and *PRO* are the individual values of the variables, $\sigma_{{FEV1_{s} }}^{2}$ and $\sigma_{{PRO_{s} }}^{2}$ are the state-specific variances of the variables (residual error), and *ρ*_*s*_ is the correlation between the variables equal to $\sigma_{{FEV1_{s} - PRO_{s} }} /\left( {\sigma_{{FEV1_{s} }} \sigma_{{PRO_{s} }} } \right)$. A correlation of − 0.33 between the observed variables was used, considering that there appears to be a moderate correlation between the outcome of disease specific questionnaires and FEV1 [14].

The stationary distribution, governing the probability to start in one state or another, and the transition probabilities, describing probabilities for a patient’s disease status to move from remission to exacerbation, were modelled using a logit function, to constrain the function between 0 and 1 in case random or covariate effects were included. The stationary distribution was described as follows:

7 $$P\left( {S_{t = 0} = R} \right) = \frac{1}{{1 + \exp \left( { - Logit\left( {\theta_{INIT} } \right)} \right)}},$$

where $Logit\left( {\theta_{INIT} } \right)$ corresponds to $\log \left( {\frac{{\theta_{INIT} }}{{1 - \theta_{INIT} }}} \right)$, and $\theta_{INIT}$ is the typical value for the probability of being in remission at the start. The probability of being in the exacerbation state at the start ($P\left( {S_{t = 0} = E} \right)$) is consequently $1 - P\left( {S_{t = 0} = R} \right).$ The transition probability matrix in this work was completely specified by:

8 $$\varPi = \left( {\begin{array}{*{20}c} {\pi_{RR} } & {\pi_{RE} } \\ {\pi_{ER} } & {\pi_{EE} } \\ \end{array} } \right),$$

where π_RE_ is the probability of transitioning to E given that the previous state was R, π_ER_ is the probability of transitioning to R given that the previous state was E, π_RR_ is the probability of remaining in R and π_EE_ is the probability of remaining in E. Note that the rows sums up to 1 and therefore only two elements of the matrix are estimated in the model, *π*_*RE*_ and *π*_*ER*_.

The transition probability from the exacerbation state to remission ($\pi_{ER}$) was modelled similarly to the stationary distribution:

9 $$\pi_{ER} = \frac{1}{{1 + \exp \left( { - Logit\left( {\theta_{{\pi_{ER} }} } \right)} \right)}}$$

The transition probability of going from remission to the exacerbation state, $\pi_{RE}$, included a treatment effect as well as a random effect, $\eta_{{\pi_{RE} }}$:

10 $$\pi_{RE} = \frac{1}{{1 + \exp \left( { - \left( {Logit\left( {\theta_{{\pi_{RE} }} } \right) + \eta_{{\pi_{RE} }} - TRT \cdot SLP} \right)} \right)}}$$

where $\theta_{{\pi_{RE} }}$ is a fixed effect parameter estimate of the probability of transitioning from the R to E state bounded between 0 and 1, $Logit\left( {\theta_{{\pi_{RE} }} } \right)$ is the logit function of $\theta_{{\pi_{RE} }}$, treatment (TRT), in this example, is an indicator of active treatment (0 or 1, indicating the presence or absence of drug) and SLP is an estimated parameter quantifying the drug effect magnitude. When drug is present, TRT∙SLP reduces the probability of transitioning from remission to the exacerbation state. For example, for a *SLP* value of 1, the probability of transitioning from remission was decreased by 62%, 60%, 46% and 15% from $\theta_{{\pi_{RE} }}$ values of 0.05, 0.1, 0.5 and 0.9, respectively. $\eta_{{\pi_{RE} }}$ is the random effect and is assumed to be normally distributed with a mean of 0 a variance of $\omega_{{\pi_{RE} }}^{2}$.

The other transition probabilities, $\pi_{RR}$, the probability of staying in remission given that the previous state was also remission, and $\pi_{EE }$ the probability of staying in the exacerbation state given that the previous state was the exacerbation state, were derived by subtracting $\pi_{RE}$ and $\pi_{ER}$ from 1, respectively.

### Data

The data structure was designed to mimic a large placebo-controlled parallel-arm trial of COPD patients on treatment (n = 250) or on placebo (n = 250). No demographic covariates were included. Data were simulated for 60 weeks with measurements of FEV1 and PRO available weekly. An alternative simulation dataset was created where FEV1 samples were collected less frequently, i.e. monthly, mimicking more realistic conditions of sparser biomarker data than PROs.

### Parameter estimation

NONMEM version 7.3.0 was used to simulate and estimate the data [15] and was executed through Perl-speaks-NONMEM (PsN) [16]. The likelihood was calculated using the forward algorithm, i.e. by summing all the probabilities of each state at each position, according to:

11 $$L_{j} = \sum\limits_{{1}}^{n=2} {P_{j} = \left( {\left( {\varphi_{R_{j - 1}} \cdot \pi_{RR} + \varphi_{E_{j - 1}} \cdot \pi_{ER} } \right) \cdot P\left( {S = R} \right)} \right) + \left( {\left( {\varphi_{R_{j - 1}} \cdot \pi_{RE} + \varphi_{E_{j - 1}} \cdot \pi_{EE} } \right) \cdot P\left( {S = E} \right)} \right)}$$

where $L_{j}$ represents the total likelihood at time $j$, $n$ the number of states, and $\varphi_{R_{j - 1}}$ and $\varphi_{E_{j - 1}}$ can be defined as $\frac{{P_{R_{j - 1}} }}{{L_{j - 1} }}$ and $\frac{{P_{E_{j - 1}} }}{{L_{j - 1} }}$, respectively. $P_{R_{j - 1}}$ is the probability of being in remission at the previous observation, $P_{E_{j - 1}}$ is the probability of being in the exacerbation state at the previous observation, and L_j−1_ is the total likelihood at the previous observation. $\frac{{P_{R_{j - 1}} }}{{L_{j - 1} }}$ and $\frac{{P_{E_{j - 1}} }}{{L_{j - 1} }}$ are, therefore, the contributions of the respective states to the total likelihood.

Maximum likelihood (ML) estimation was performed using the stochastic approximation expectation maximization (SAEM) algorithm followed by an importance sampling step to obtain a stable objective function value (OFV) in NONMEM. The settings used included a maximum number of iterations of 400, a termination test and other specifications as found most appropriate (NONMEM model code available in Online Appendix 1). Parameters in the model were expressed using MU-referencing in NONMEM where parameters in the model are associated with IIV linearly, improving the efficiency of expectation maximization (EM) algorithms. When suitable, to evaluate the most likely hidden states chain, the Viterbi algorithm was run, through a post hoc subroutine (hmm.f90) made available as part of NONMEM [17]. The Viterbi algorithm works recursively by using the individual likelihoods computed for the model in combination with the sequences of observations [18]. The most probable sequence is obtained when the likelihood of a sequence ceases to increase, in comparison to other explored sequences. The Viterbi algorithm is used to reduce the computational burden of having to evaluate all possible hidden state sequences in an individual as only the most likely sequences up to the current observation are kept in the calculation. Implementation and estimation of a HMM in NONMEM does not require any specific subroutine. The user defines the transition probability matrix, initial state conditions and the emission probabilities. During ML estimation, the system is first initialized according to the initial state conditions and emission probabilities. Then, for each subsequent observation time, the system is estimated according to Eq. 11. The provided NONMEM code is annotated to describe these aspects.

### Accuracy and precision

The stochastic simulation and estimation (SSE) functionality in PsN was used to obtain parameter precision and accuracy [19]. The SSE is a two-step method where first a number of data sets (here 100 per scenario) were simulated using the model of interest and subsequently estimated with the same model. The resulting parameter precision and accuracy of parameters was then calculated, with the results being summarized numerically through the relative root mean squared error (RRMSE):

12 $$RRMSE\left( \% \right) = 100 \sqrt {\frac{1}{N} \cdot \mathop \sum \limits_{i} \frac{{(estimated_{i} - true_{i} )^{2} }}{{true_{i}^{2} }}} ,$$

where true values are defined as the parameter values set in the simulation model.

Parameter values were varied to investigate the effect of parameter magnitude on the imprecision and accuracy of the estimates resulting in 14 model scenarios (Table 2). Focus was on the parameters influencing the transition probabilities (including the magnitude of drug effect and IIV) and on the correlation in the bivariate model, as estimation of these parameters may influence other parameters in the model as well.

**Table 2:** Parameter precision was evaluated by running stochastic simulation and estimation (samples = 100) with 14 different scenarios

Parameters subject to change	Reference scenario	Scenario exploring effect of transition probabilities magnitude only	Scenarios exploring effect of drug effect magnitude				Scenarios exploring effect of inter individual variability magnitude				Scenarios exploring effect of correlation magnitude		Scenarios exploring trial design	
	1	2	3	4	5	6	7	8	9	10	11	12	13	14
*SLP*	1.00	1.00	**2.00**	**0.50**	**2.00**	**0.50**	1.00	1.00	1.00	1.00	1.00	1.00	1.00	1.00
$\theta_{{\pi_{RE} }}$	0.05	**0.10**	0.05	0.05	**0.10**	**0.30**	0.05	0.05	**0.10**	**0.10**	0.05	**0.10**	0.05	**0.10**
$\theta_{{\pi_{ER} }}$	0.15	**0.30**	0.15	0.15	**0.30**	**0.30**	0.15	0.15	**0.30**	**0.30**	0.15	**0.30**	0.15	**0.30**
$\omega_{{\pi_{RE} }}^{2}$	0.06	0.06	0.06	0.06	0.06	0.06	**0.00**	**0.12**	**0.00**	**0.12**	0.06	0.06	0.06	0.06
$\rho_{R}$ and $\rho_{E}$	− 0.33	− 0.33	− 0.33	− 0.33	− 0.33	− 0.33	− 0.33	− 0.33	− 0.33	− 0.33	− **0.66**	− **0.66**	− 0.33	− 0.33
Number FEV1 samples, number PRO samples	60, 60	60, 60	60, 60	60, 60	60, 60	60, 60	60, 60	60, 60	60, 60	60, 60	60, 60	60, 60	**15, 60**	**15, 60**

Bold parameters indicate changed parameters from the reference scenario

*SLP* drug effect, π_RE_ transition from remission to exacerbation, π_ER_ transition from exacerbation to remission, $\omega_{{\pi_{RE} }}^{2}$ IIV of *π*_*RE*_, *ρ*_*R*_ and *ρ*_*E*_ correlation between FEV1 and PRO in the states

### Ignoring correlation in the model

To determine the effect of ignoring the correlation when present in the simulations, a separate SSE analysis was performed, where the simulation model included a correlation while a reduced estimation model without correlation, in addition to the full simulation model, was employed.

The bivariate likelihood without correlation was expressed as:

13 $$P\left( {Y_{FEV1} ,Y_{PRO} |S = s} \right) = \frac{1}{{2\pi \sqrt {\sigma_{{FEV1_{s} }}^{2} \sigma_{{PRO_{s} }}^{2} } }} \cdot e^{{ - \frac{1}{2}\left( {\left( {\frac{{Y_{FEV1} - FEV1_{s} }}{{\sigma_{{FEV1_{s} }} }}} \right)^{2} + \left( {\frac{{Y_{PRO} - PRO_{s} }}{{\sigma_{{PRO_{s} }} }}} \right)^{2} } \right)}} .$$

The misfit of ignoring the correlation when it was present was quantified with the average ΔOFV, which was calculated by subtracting the OFV of each of the 100 reduced models from the OFV from each of the corresponding 100 full models and then taking the mean (ΔOFV = OFV_reduced_ − OFV_full_).

### Power to detect a drug effect

To assess the power to detect a drug effect and to determine differences in power between univariate and bivariate models, the Monte-Carlo mapped power (MCMP) methodology was used [20]. According to the general MCMP steps, a large simulated dataset (5000 individuals) was simulated from a model with a treatment effect reducing the probability to transition from remission to exacerbation. The whole dataset was then estimated with a full model, including the treatment effect, and a reduced model, without any treatment effect. A likelihood ratio test (LRT) was then applied, using the differences between the sum of the individual OFVs (iOFVs). The iOFVs were resampled 10,000 times for each sample size of interest, and the sum of iOFVs for each sample was calculated. The percentage of sum of iOFVs greater than the significance criterion (α = 0.05) was taken as the power for the specific sample size, resulting in a full power versus sample size curve. Three scenarios, based on the reference model (scenario 1, Table 2) were evaluated where SLP was set to 1, 0.5 or 2. An additional scenario was also evaluated to determine whether monthly FEV1 samples in a bivariate model would improve the power to detect a drug effect over a univariate model only considering PRO samples (the less informative variable). Here, SLP was set to 1.

When only one endpoint was simulated (throughout or at certain time points), only a univariate model for emission probabilities was necessary. It was expressed as (for example for PRO):

14 $$P\left( {Y_{PRO} |S = s} \right) = \frac{1}{{2\pi \sqrt {\sigma_{{PRO_{s} }}^{2} } }} \cdot e^{{ - \frac{{\left( {Y_{PRO} - PRO_{s} } \right)^{2} }}{{2\sigma_{{PRO_{s} }}^{2} }}}} .$$

## Results

### Model

**Fig. 1:**
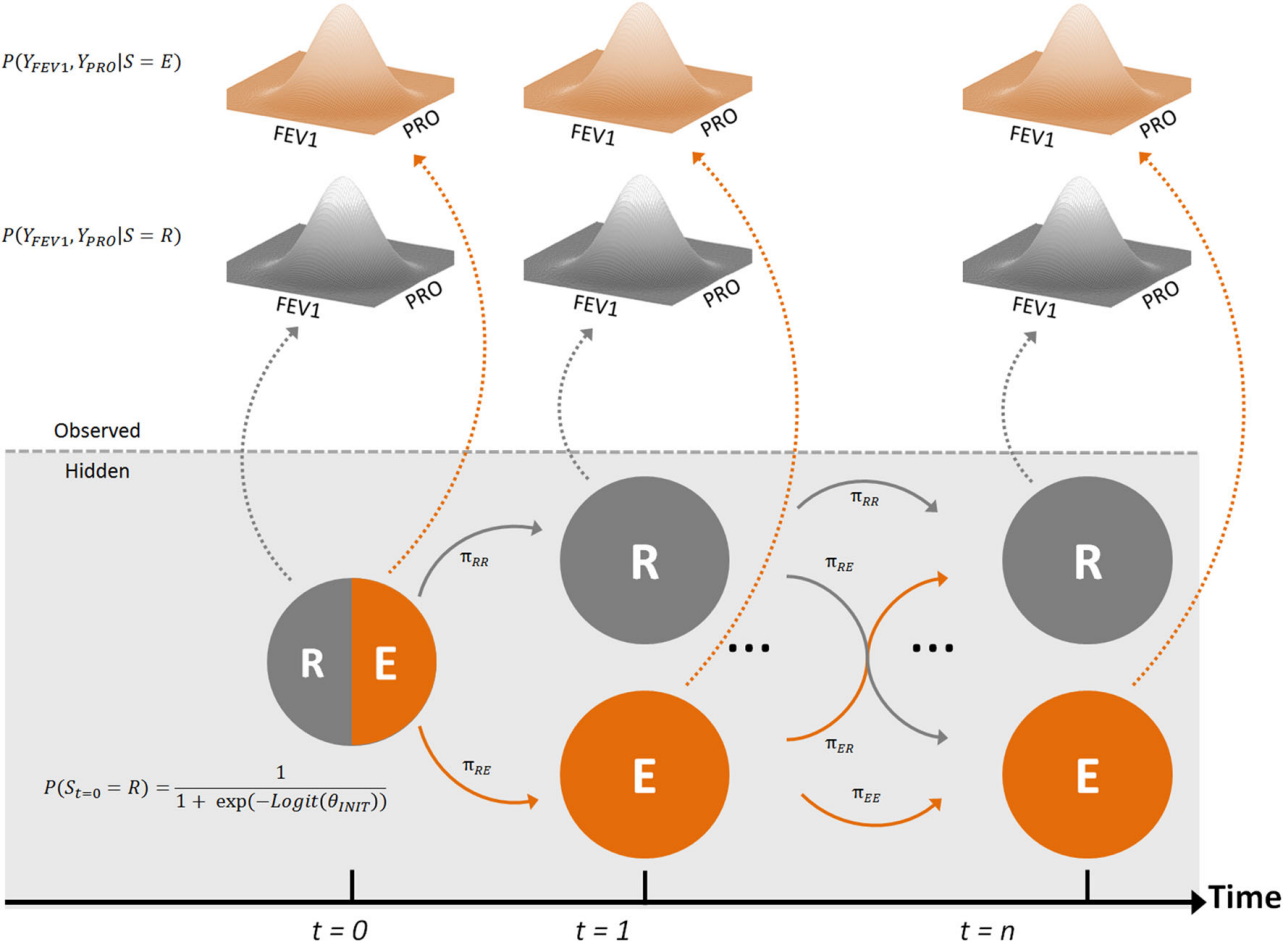
A schematic representation of the bivariate hidden Markov model used in this work. Two observation sources, FEV1 measurements and PROs, depend on remission (R, grey) and exacerbation (E, orange) states. The dashed horizontal grey line separates hidden features in the mode from observable ones. The observations are modeled using a bivariate Gaussian function. Transition parameters govern the probability of transitioning from remission to the exacerbation state (π_RE_), transitioning from the exacerbation state to remission (π_ER_), or staying in the respective states (π_RR_ and π_EE_). Dashed arrows represent the emission of observations from the hidden states. At the first time point (denoted t = 0) the state in which the system starts from is dictated by the initial state probability (Color figure online)

A bivariate MHMM was developed by combining the two univariate models through bivariate Gaussian functions (schematically represented in Fig. 1). The model considered two correlated observations, FEV1 and PRO, arising from one of two underlying states, representing the remission (R) and an exacerbation (E) states (Eqs. 1–9).

**Fig. 2:**
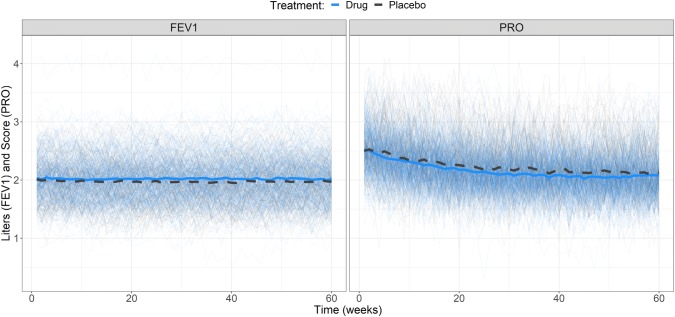
Simulations of FEV1 (left panel) and PRO (right panel) from the bivariate hidden Markov model colored by treatment status (drug = blue, placebo = dark grey). The thick blue solid line and dark grey dashed line are the means of the observations under drug or placebo treatment, respectively (Color figure online)

**Fig. 3:**
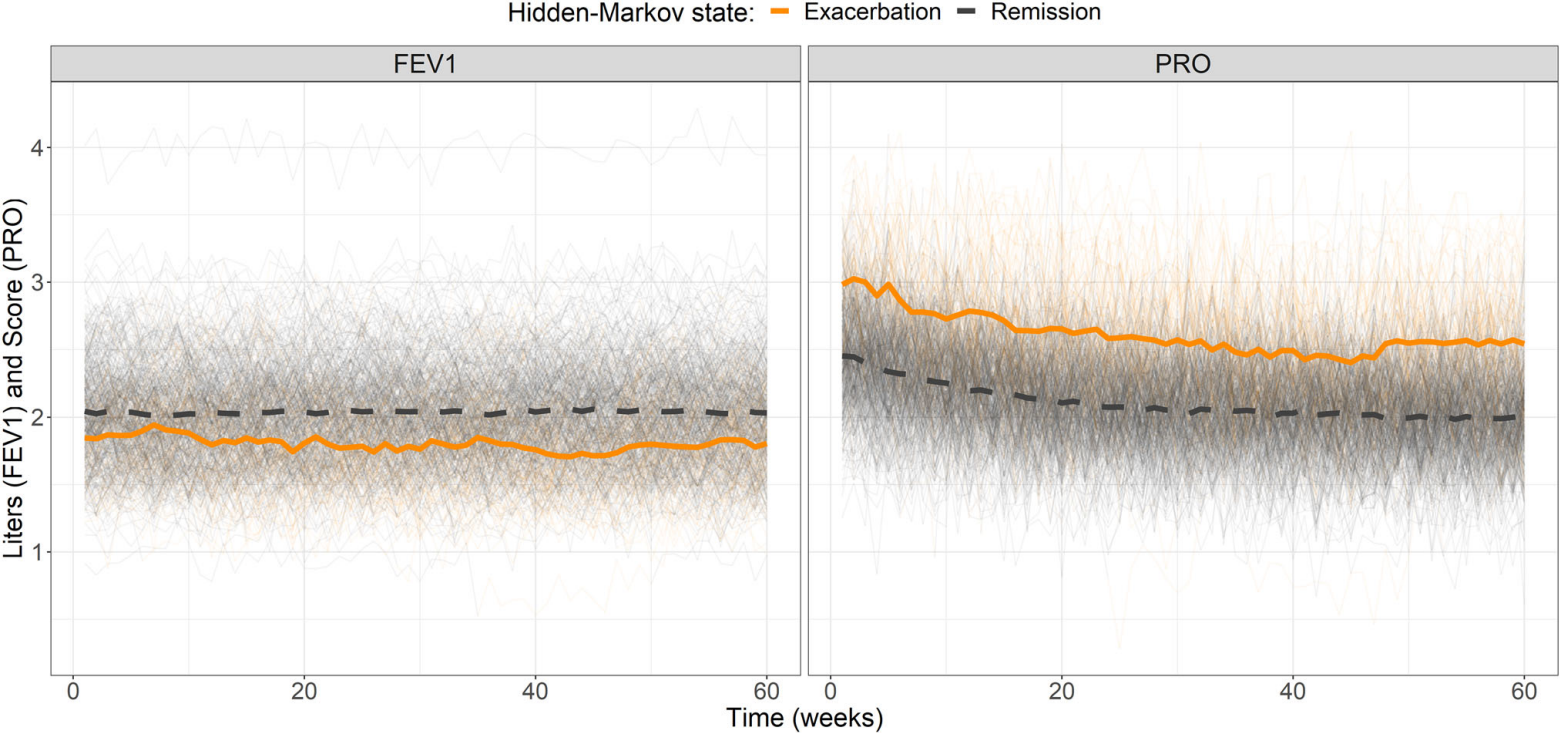
Simulations of FEV1 (left panel) and PRO (right panel) from the bivariate hidden Markov model. Dark grey and orange lines are observations from remission and exacerbation states, respectively. The thick dark grey dashed line and orange solid line are the means of the observations coming from the latent and active disease states, respectively (Color figure online)

**Fig. 4:**
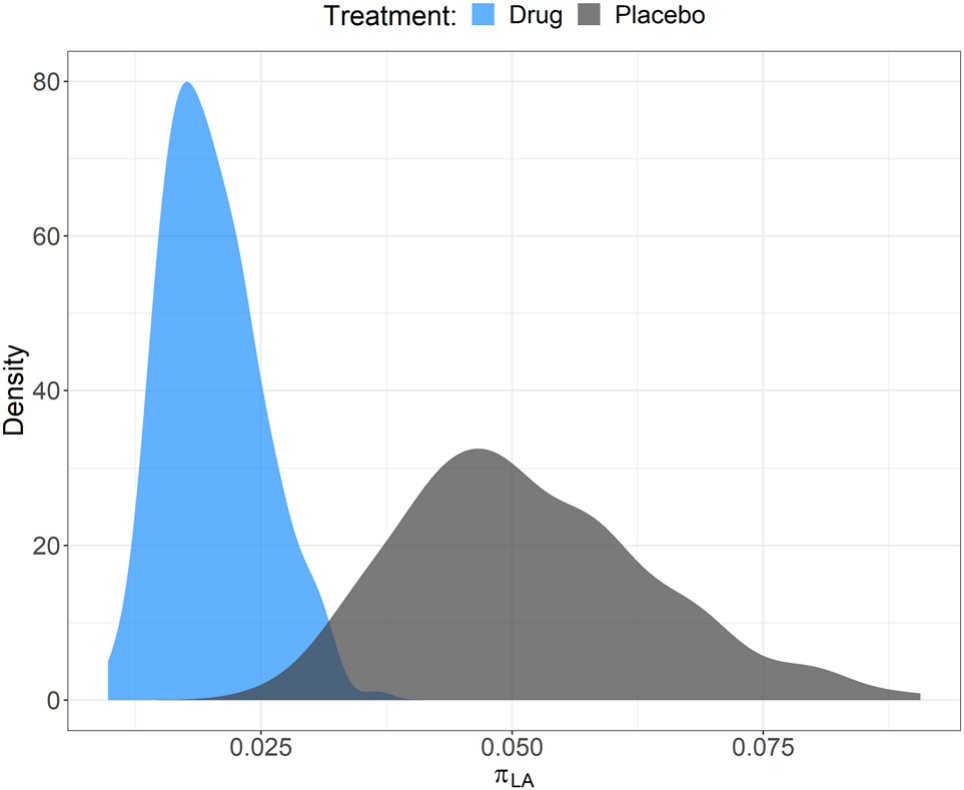
Individual values for the transition rate from remission to the exacerbation state (π_RE_) on drug (blue) or placebo (dark grey) (Color figure online)

Simulations illustrating the influence of the drug effect and state sequence from the base model are presented in Figs. 2 and 3, respectively. The drug effect on π_RE_ is presented in Fig. 4. There was an obvious difference between the two states and *π*_*ER*_ was lower in patients who received treatment (*SLP* = 1) resulting in small, but noticeable, differences in FEV1 and PRO (Figs. 2 and 4).

### Parameter estimation

**Fig. 5:**
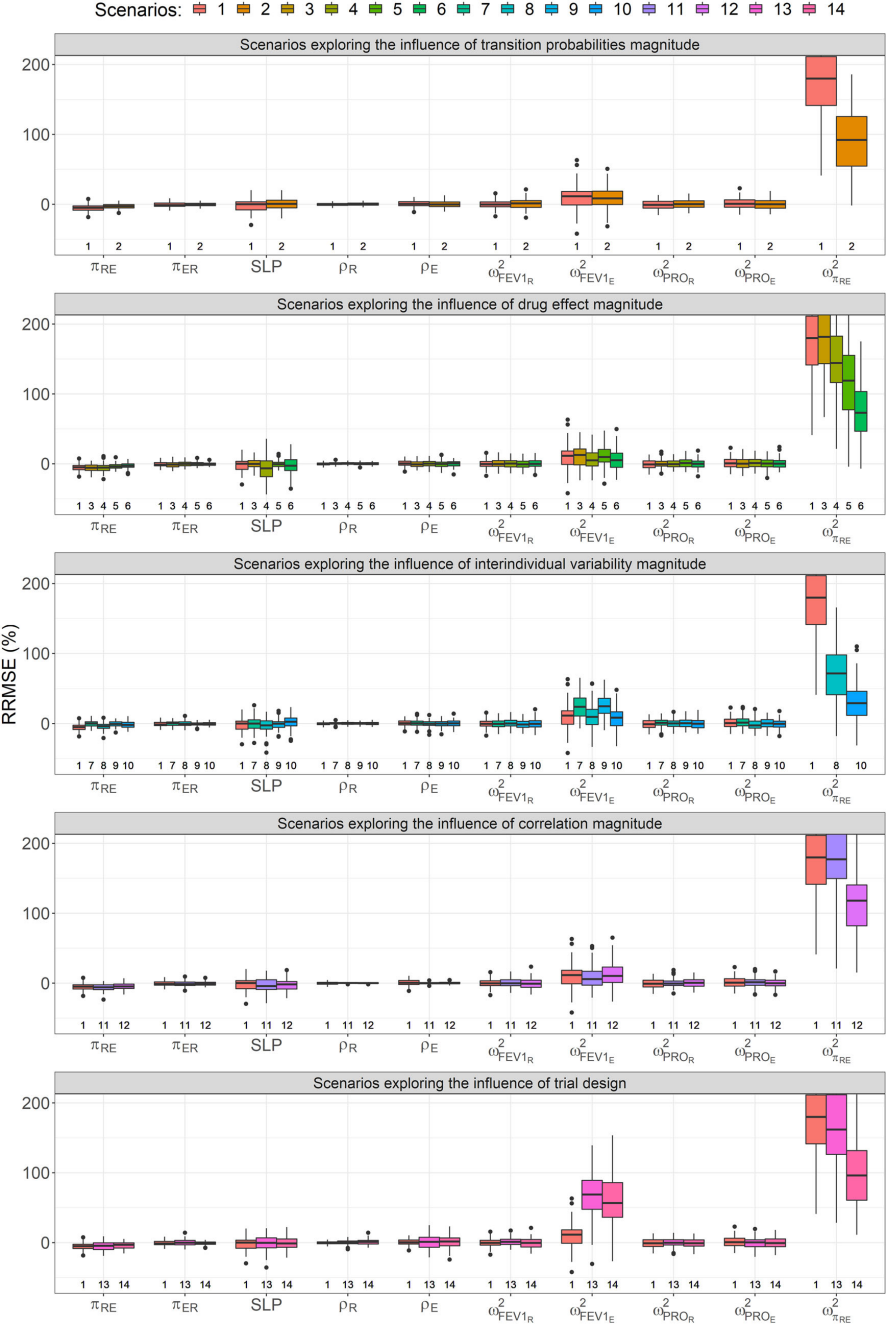
Relative root mean squared error (y-axis) of selected parameters (x-axis) and explored scenarios. The presented parameters are the transition probability from remission to exacerbation ($\pi_{RE}$), the transition probability from the exacerbation state to remission ($\pi_{ER}$), the drug effect (*SLP*), correlations in remission and the exacerbation states (*ρ*_*R*_ and *ρ*_*E*_, respectively), the variance of FEV1 in the remission and exacerbation states ($\omega_{{FEV1_{R} }}^{2}$ and $\omega_{{FEV1_{E} }}^{2}$, respectively), the variance of PRO in the remission and exacerbation states ($\omega_{{PRO_{R} }}^{2}$ and $\omega_{{PRO_{E} }}^{2}$, respectively) and the variance of $\pi_{RE}$ ($\omega_{{\pi_{RE} }}^{2}$). Scenario 1 is included as a comparison in all tested scenarios. Numbers indicate scenarios

Results with regards to parameter precision are presented in Fig. 5 and in Online Appendix 2. Scenario 1 (reference scenario) resulted in relatively well estimated parameters (RRMSE ≤ 10%) with the exception of $\omega_{{FEV1_{E} }}^{2}$ and $\omega_{{\pi_{RE} }}^{2}$ which were estimated with RRMSEs of 19.5% and 187%, respectively. In general, parameters estimated with the evaluated scenarios were precisely estimated without large bias, apart from *SLP*, $\omega_{{FEV1_{E} }}^{2}$ and $\omega_{{\pi_{RE} }}^{2}$, with the latter being consistently the most inaccurately and imprecisely estimated parameter (Fig. 5). Doubling the transition probabilities, *π*_*RE*_ and *π*_*ER*_, from 0.05 and 0.15 to 0.1 and 0.3, respectively, moderately improved the estimation of all parameters in all scenarios, reducing the average RRMSE across all parameters by 5%.

In scenario 4, where the drug effect was 0.5, the RRMSE of *SLP* was 17.6% and improved with increasing parameter magnitude of *SLP* (RRMSE = 10.0% and 6.7% for scenarios 1 and 3, respectively). A similar trend was observed for the relative bias of *SLP,* which was greatest when *SLP* was low and improved with increasing parameter magnitude. Doubling the transition probabilities (scenario 5) decreased the RRMSE of *SLP* in the investigations of the influence of drug effect magnitude by between 2.1 and 5.2 percentage points.

When no IIV was present on *π*_*RE*_ (Scenario 7) the estimation of parameters in the model was relatively precise (RRMSE < 10%) and unbiased with the exception of $\omega_{{FEV1_{E} }}^{2}$ (RRMSE = 29.7%). Scenario 1 showed a slight bias in *π*_*RE*_ which was not present in Scenario 7 and RRMSE of *π*_*RE*_ decreased from 7.2 to 4.7% when no IIV was present on the parameter. Increasing the magnitude of $\omega_{{\pi_{RE} }}^{2}$(scenario 8) resulted in > 100 percentage point drop in the RRMSE of that parameter compared with scenario 1.

Correlation had a negligible effect on parameter precision of parameters other than ρ_R_ and ρ_E_, which were more accurately estimated with a larger negative correlation. The RRMSE of *ρ*_*R*_ and *ρ*_*E*_ decreased from 1.9 and 4.7 to 0.6 and 1.3%, respectively, when comparing scenarios 1 and 11. In general the correlation between the variables was accurately (average RRMSE < 5% in tested scenarios) estimated.

Reducing the number of available FEV1 samples resulted in a small increase in RRMSE for most parameters in the model. The largest increase in RRMSE was observed for $\omega_{{FEV1_{E} }}^{2}$ which increased from 19.5% in scenario 1 to 74.7% in scenario 13.

**Fig. 6:**
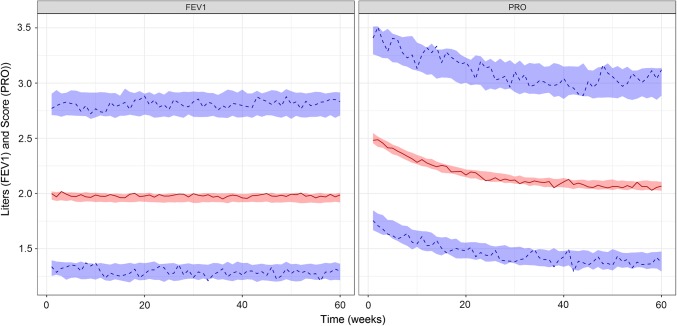
Visual predictive check of scenario 1. The red solid line and the blue dashed lines indicate the observed median and 97.5th and 2.5th percentiles of the observed data, respectively. The shaded regions are the 95% confidence interval of simulations from the model estimated in scenario 1 (Color figure online)

Simulations from the model estimated (Fig. 6) in scenario 1 illustrated that despite the apparent bias in $\omega_{{\pi_{RE} }}^{2}$ the model still performed well as the observed percentiles fell within the simulated confidence interval corresponding to those percentiles (based on 100 simulations).

### Correlation in the model

Estimation of data simulated with *ρ*_*R*_ and *ρ*_*E*_ = − 0.33 with a reduced model (both parameters set to zero) and a full model (*ρ*’s estimated) resulted in an average ΔOFV of 2993 when the simulation model was based on scenario 1. Estimation of data simulated with *ρ* = 0 with a reduced model and a full model resulted in an average ΔOFV of − 2.11, an insignificant difference between the models.

### Power to detect a drug effect

**Fig. 7:**
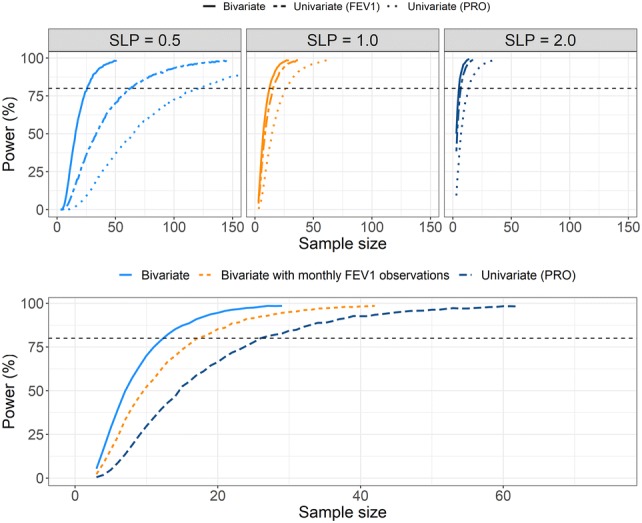
Power to detect a linear drug effect (SLP) of different magnitudes (top panels). The different line types indicate which model was used to detect the drug effect. The horizontal dashed line indicates 80% power. The bottom panel shows the power to detect a linear drug effect (*SLP *= 1) given a bivariate model with weekly observations of both PRO and FEV1, a bivariate model with weekly PRO observations and monthly FEV1 observations and a univariate considering only weekly PRO observations

The power to detect a hypothetical drug effect was estimated for three different values of *SLP*, 0.5, 1.0 and 2.0. The general trend was that the bivariate model was more powerful than either univariate models, with the univariate model for FEV1 observations being more powerful than the model for PRO observations. Further, the lower the drug effect the more subjects were needed for equal power. When *SLP* = 0.5, 80% power was achieved with ~ 25 subjects for the bivariate model compared with ~ 63 subjects using the FEV1 model and > 100 subjects using the PRO model (Fig. 7, left panel). When *SLP* = 1, the number of individuals required for 80% power was ~ 5 subjects in the bivariate model compared with ~ 7 subjects in the FEV1 model and ~ 13 subjects in the PRO model.

The power to detect a drug effect (*SLP* = 1) was, for the same number of individuals, increased by adding monthly FEV1 observations compared to the univariate model for PRO alone, decreasing the number of subjects needed for 80% power from ~ 26 to ~ 17 (Fig. 7, right panel).

## Discussion

Pharmacometric models may be used to analyse the time-course of a disease, which is lost when using simple parametric models that only consider the data at the end of the study. More complex models in this family, accounting for several different observation sources, increase the amount of information available for disease classification. In this work, a bivariate MHMM was developed for simulating and analysing hypothetical COPD data consisting of PROs and FEV1 measurements collected weekly for 60 weeks.

The example disease, COPD, was used because of the latent nature of exacerbations, but the model can be used on any data where inference about a latent disease state is of interest. The classification of COPD severity is complex and is often based on the number of exacerbations that a patient reports. However, summarizing exacerbation frequency is difficult since exacerbations are frequently underreported and long periods of remission can precede and succeed relatively brief periods of exacerbation [21].

The model assumptions that were made were deemed to be acceptable in light of the goal of the work as follows. When simulating with the developed model it was assumed that both PRO and FEV1 were continuous and normally distributed in the population regardless of which state an individual was in. However, the variables in the developed model need not be normally distributed and the model could simulate any distribution of interest, provided that there is a mathematical function describing the distribution. A normal distribution was assumed for simplicity especially considering that we aimed to correlate the variables through a function, i.e. the multivariate Gaussian function. We included random effects on the modes (IIV), variances of the observed variable distributions (residual error) and assumed distinct distributions of PRO and FEV1 for each disease state. We also assumed that the variance of PRO was greater than the variance of FEV1 given the uncertain nature of patient reported end points compared with biomarker data [22, 23]. Since PROs reflect patients’ perspective including tolerance towards certain effects, large within-patient variability can be expected depending on the mind-set of the patient at the time of the PRO evaluation. Further, the IIV of PRO was larger than for FEV1, assuming a more variable response between individuals for PRO than for FEV1, which is reasonable given inconsistency in people’s perception. In this work the initial probability of being in remission was set to 90%, assuming that most individuals, when starting the study, were in remission. Given that exacerbation times can vary markedly (durations between 1 and ~ 200 days have been reported [21]), the number 90% was chosen arbitrarily but thought to be plausible based on that the inclusion criteria for two large scale studies in COPD, TRISTAN and ISOLDE, was a history of at least one or two exacerbations in the past year, respectively [24, 25]. Since PROs were not simulated to represent any known tools, arbitrary values were chosen. The assumption was also made that PROs decreased with time, indicating a hypothetical PE with a maximum effect of 20%, a high value compared to the ~ 6% reported in a systematic review [26]. Moreover, in the cited review, the PE could not be clearly separated from bias. However, the main aim of this work was to develop a bivariate MHMM and determine the parameter estimation properties of said model, not to reconstruct a previously observed clinical scenario. With a treatment effect of 1, a very small change in the average PRO score and FEV1 values was observed. The difference in FEV1 between the placebo group and treatment group is consistent with the small differences frequently observed in clinical trials of drugs for COPD [27–29]. However, it may be expected that a larger difference would have been observed in PROs. This could have been included in the model with two treatment effects, but was not considered for simplicity.

Using HMMs practically requires the solution of three distinct problems; (1) obtaining the likelihood of the observations given an HMM, (2) finding the most probable state sequence given an observation sequence and (3) given the HMM and an observation sequence obtain the best parameter estimates in the HMM. The third problem, also known as the learning problem, is the focal point of this study, since the observation sequence is simulated based on a state sequence from the HMM and thus known. In real data cases, where the underlying state sequence is not known, the practical approach is to develop a HMM that may describe the system (with sufficient number of states and observation types) estimate it and then use it to obtain the most likely state sequence given the observations. Expectation maximization (EM) algorithms can be used to solve the learning problem and are readily available in several software packages [30], we therefore used SAEM in this analysis.

Parameter precision was relatively high for all parameters in the model in all tested scenarios apart from $\omega_{{\pi_{RE} }}^{2}$. Even when the magnitude of the parameter was increased two-fold it was relatively biased and imprecise. Separating IIV on hidden states parameters from IIV on parameters describing the observed variables represents a challenge. In general, IIV on transition probabilities and the initial state probability may be difficult to estimate. In fact, it may be impossible to determine whether the variability in the observed variables is acquired from variability in “hidden state parameters” or other. Despite the issues with estimating $\omega_{{\pi_{RE} }}^{2}$, model simulations from the model in scenario 1 were descriptive of the observed data.

Parameters estimated in the model benefited with regards to precision when more transitions occurred in the data (i.e. when the transition rates were doubled). With few transitions the amount of information on latent variables in the system is limited and thus, for models such as the one developed in this work, transitions between the states of interest are necessary for the estimation of parameters. However, the dependence on number of transitions likely depends on the difference in the observed variables in the two modelled states, where larger differences should enable more precise estimation of “hidden state parameters”.

The drug effect was relatively well estimated in the tested scenarios, but precision improved with increasing magnitude, as expected. This may have implications for the analysis of the data using MHMMs, which may require certain magnitudes of expected drug effects to be viable, although these results suggest that small differences in the observed variables due to a drug effect are detectible even with small sample sizes. The drug effect in this model influences just one of the transition rates and differences in the observed variables propagate from there. It may be possible to include a drug effect on both the transition probabilities and the observed variables. For instance, a disease modifying drug effect could be incorporated on the transition probabilities while a symptomatic drug effect could be added on an observed variable such as PRO. Additional studies are needed to determine whether it is possible to identify two or more drug effects present on observed and hidden variables in the model.

Correlation had little overall effect on the precision of parameter estimates as results with low and high negative correlations were very similar. However, our results indicate that not considering correlation when it is present results in a worse overall fit than when it is considered, advocating for the use of a multivariate model. If observations of two, or more, variables are collected to infer about the disease state in a patient, it may be natural to assume that they are correlated. If they were not correlated, they would have little value for inference of the hidden state sequence for instance. In this work, we assume that the correlations are equal in both underlying states. This assumption may be relaxed, which would make the model more flexible. Most variables may be either positively correlated or negatively correlated over all hidden states, these constraints in the model may be more mechanistic than allowing correlations to vary freely. When data were simulated assuming no correlation and estimation allowed the estimation of a correlation the results indicated no difference between the full and reduced models, which is expected since the models are nested. Some instability was identified in the model which was estimated using SAEM in NONMEM with a secondary importance sampling step to obtain the OFV of the final set of parameters. A parallel run (with 5 retries) with scenario 1 parameter estimates resulted in an OFV fluctuation of approximately 5 points.

The power to detect a drug effect included on the transition probability going from remission to exacerbation ($\pi_{RE}$) in the model was higher with the bivariate model than with either two univariate model. The number of observations entering the bivariate model is twice of the univariate models and, thus, the bivariate model makes use of more information about the drug effect. When the drug effect was low (SLP = 0.5) the bivariate model reduced the number of subjects resulting in an 80% power to detect a drug effect by approximately 78% over a univariate model considering only PRO observations. The number of subjects required for detection of a drug effect in this analysis was relatively low and is expected to be larger for observed variables associated with larger uncertainty but the results show that the use of a bivariate model to detect drug effects is advocated, when possible, over using single variable models.

## Conclusion

In this work, a bivariate MHMM was developed for simulating and analysing correlated continuous observations connected to hidden states. The data generated consisted of PROs and FEV1 measurements in COPD patients conditional on latent/hidden exacerbation/remission disease states. Parameters associated with the “observable” portion of the model were in general more precisely estimated than those associated with the “hidden” portion; in addition, precision depended on the magnitude of parameters such as the transition probabilities, the drug effect on the transition probabilities, IIV of the transition probability and correlation. The power to detect a hypothetical drug effect was consistently highest with the bivariate model compared with univariate models.

## Electronic supplementary material

Below is the link to the electronic supplementary material.

Supplementary material 1 (PDF 63 kb)

Supplementary material 2 (PDF 396 kb)
