# Supplementary material for: Handling underlying discrete variables with bivariate mixed hidden Markov models in NONMEM
Source: J Pharmacokinet Pharmacodyn. 2019 Oct 26;46(6):591–604. doi: 10.1007/s10928-019-09658-z (PMC6868114; doi:10.1007/s10928-019-09658-z)
Supplement: Supplementary file 1 — Supplementary material 1 (PDF 63 kb) [file 10928_2019_9658_MOESM1_ESM.pdf]

Appendix 1

```
$PROBLEM    est400

$INPUT      ID TIME DV DV2 TRT ; DV is FEV1 and DV2 is PRO

$DATA       simtab400 IGNORE=@

$ABBREVIATED DECLARE GAMMA(2,2),DELTA(2),PO(2),PR(2),POINTS(3600,2)

; GAMMA is the transition probability matrix, DELTA are the initial state probabilities, PR are the
emission probabilities. PO and POINTS are required for the Viterbi algorithm (requires a modified
model code, see reference 17)

$PRED

; Dependent variable 1, FEV1.

MU_1 = THETA(1) ; mode of FEV1, state 1 (S1)

MU_2 = THETA(2) ; mode of FEV1 in state 2 (S2) to be subtracted from FEV1 in state 1.

VAR1S1 = LOG(MU_1)

VAR1S2 = LOG(MU_2)

LAM1 = EXP(VAR1S1 + ETA(1))

LAM2 = LAM1 - EXP(VAR1S2 + ETA(2)) ; Model specific, in this case we subtract FEV1 in S2 from S1 to
get its value in S2.


; FEV1 SIGMAS

MU_9 = THETA(9) ; Variance (residual error) of FEV1 in S1

SIGMA1S1 = MU_9 + ETA(9)

MU_10 = THETA(10)

SIGMA1S2 = MU_10 + ETA(10) ; Variance (residual error) of FEV1 in S2


; Dependent variable 2, PRO

MU_3 = THETA(3) ; mode of PRO, S1

MU_4 = THETA(4) ; mode of PRO in S2 to be added to PRO in S1.

VAR2S1 = MU_3 + ETA(3)

PRO1 = VAR2S1 * (1 - 0.2 * (1-EXP(-(LOG(2)/10*TIME)))) ; Placebo effect decrease by TIME in PRO in
S1

VAR2S2 = MU_4 + ETA(4)

PRO2 = PRO1 + VAR2S2 ; Model specific, in this case we add PRO in S2 to S1 to get its value in S2.


; PRO SIGMAS

MU_11 = THETA(11) ; Variance (residual error) of PRO in S1

SIGMA2S1 = MU_11 + ETA(11)

MU_12 = THETA(12) ; Variance (residual error) of PRO in S2

SIGMA2S2 = MU_12 + ETA(12)


;Stationary distribution, probability to start in S1 or S2

MU_5 = THETA(5)

LOGIT_MU5 = LOG(MU_5/(1-MU_5))
```

```
DELTA1 = EXP(LOGIT_MU5+ETA(5))/(1 + EXP(LOGIT_MU5+ETA(5)))

DELTA(1) = DELTA1 ; Probability of starting in S1 (was logit transformed to ensure 0 > values > 1.

DELTA(2) = 1.0 - DELTA(1) ; Probability of starting in S2 is 1 – PR(S1_0)


; Transition probabilities, i.e. move between S1 and S2

MU_6 = THETA(6)

LOGIT_MU6 = LOG(MU_6/(1-MU_6))

MU_7 = THETA(7)

LOGIT_MU7 = LOG(MU_7/(1-MU_7))


; Drug effect (SLP), TRT indicator

MU_8 = THETA(8) ; drug effect on the transition probability from S1 to S2.

SLOPE = MU_8 + ETA(8)

GAMMA(1,2) = EXP(LOGIT_MU6+ETA(6)-TRT*SLOPE)/(1 + EXP(LOGIT_MU6+ETA(6)-TRT*SLOPE)) ; Probability of
transitioning from S1 to S2.

GAMMA(2,1)  = EXP(LOGIT_MU7+ETA(7))/(1 + EXP(LOGIT_MU7+ETA(7)))  ; Probability of transitioning from
S2 to S1.

GAMMA(1,1) = 1.0 - GAMMA(1,2) ; Probability of staying in S1

GAMMA(2,2) = 1.0 - GAMMA(2,1) ; Probability of staying in S2



; Bivariate probability density function governing emission probabilities

; State = Remission (state 1)

MU_13 = THETA(13) ; Correlation between FEV1 and PRO in S1.

RHOS1 = MU_13 + ETA(13)

EXP1S1 = (DV - LAM1)/SQRT(SIGMA1S1)

EXP2S1 = (DV2 - PRO1)/SQRT(SIGMA2S1)

EXP3S1 = -2*RHOS1*EXP1S1*EXP2S1

DETS1 = SIGMA1S1*SIGMA2S1*(1-RHOS1**2)  ; Determinant when state = 1


; State = Exacerbation (state 2)

MU_14   = THETA(14) ; Correlation between FEV1 and PRO in S2.

RHOS2 = MU_14 + ETA(14)

EXP1S2 = (DV - LAM2)/SQRT(SIGMA1S2)

EXP2S2 = (DV2 - PRO2)/SQRT(SIGMA2S2)

EXP3S2 = -2*RHOS2*EXP1S2*EXP2S2

DETS2 = SIGMA1S2*SIGMA2S2*(1-RHOS2**2)  ; Determinant when state = 2


PIE     = 3.14159265


PR(1) = 1/(2*PIE*SQRT(DETS1)) * EXP(-1/(2*(1-RHOS1**2)) * (EXP1S1**2 + EXP3S1 + EXP2S1**2))
```

```
PR(2) = 1/(2*PIE*SQRT(DETS2)) * EXP(-1/(2*(1-RHOS2**2)) * (EXP1S2**2 + EXP3S2 + EXP2S2**2))


; Likelihood

IF(NEWIND.NE.2)THEN   ; at the first observation time point

PHI1=0.0

PHI2=0.0

V1=DELTA(1)*PR(1)

V2=DELTA(2)*PR(2)

ENDIF


IF(NEWIND.EQ.2)THEN  ; subsequent observations

PHI1=PHI1

PHI2=PHI2

; V1 and V2 are the likelihoods of the observations from S1 and S2, respectively. For more details
please refer to equation 11 in the manuscript.

V1=(PHI1*GAMMA(1,1)+PHI2*GAMMA(2,1))*PR(1)

V2=(PHI1*GAMMA(1,2)+PHI2*GAMMA(2,2))*PR(2)

ENDIF


LIKE= V1+V2

IF(LIKE<1.0E-100) LIKE=1.0E-100

Y=LIKE

PHI1=V1/LIKE ; Contribution of observations resulting from S1 to the total likelihood

PHI2=V2/LIKE ; Contribution of observations resulting from S2 to the total likelihood



; Fixed effects parameters for dependent variables

$THETA  (0.000001,2.00) ; FEV1 in remission, S1 (2)

$THETA  (0.000001,0.25) ; Subtract from FEV1 when in exacerbation, S2 (0.25)

$THETA  (0.000001,2.50) ; PRO in remission, S1  (2.5)

$THETA  (0.000001,0.50) ; Subtract from PRO when in exacerbation, S2 (0.5)


; Hidden state parameters

$THETA  (0.000001,0.9,0.9999999) ; The probability of being in remission at Time = 0 (0.9)

$THETA   (0.000001,0.1,0.9999999)  ;  piRE,  the  transition  probability  from  remission  (S1)  to
exacerbation (S2) (0.1)

$THETA   (0.000001,0.3,0.9999999)  ;  piER,  the  transition  probability  from  exacerbation  (S2)  to
remission (S1)  (0.3)

$THETA  1 ; SLP (TRT), treatment effect (1)


; Variance (residual error)
```

```
$THETA  (0.000001,0.015) ; variance of residual error for FEV1 in S1 (0.015)

$THETA  (0.000001,0.015) ; variance of residual error for FEV1 in S2 (0.015)

$THETA  (0.000001,0.05)  ; variance of residual error for PRO in S1 (0.05)

$THETA  (0.000001,0.05)  ; variance of residual error for PRO in S2 (0.05)


; Correlation

$THETA (-0.99999,-0.33,0.99999) ; rhoR, correlation between the variables in S1 (-0.33)

$THETA (-0.99999,-0.33,0.99999) ; rhoE, correlation between the variables in S2 (-0.33)


$OMEGA

; Interindividual variability parameters for dependent variables

0.03  ; FEV1R

0.03  ; FEV1E

0.09  ; PROR

0.09  ; PROE

; Interindividual variability parameters for dhidden state

0 FIX ; init (0)

0.06  ; piRE

0 FIX ; piER

0 FIX ; SLP (0)

; Variance

0 FIX ; sigma1s1 (0)

0 FIX ; sigma1s2 (0)

0 FIX ; sigma2s1 (0)

0 FIX ; sigma2s2 (0)

; Correlation

0 FIX ; rhoR (0)

0 FIX ; rhoE (0)


$ESTIMATION METHOD=SAEM LAPLACE LIKE NITER=400 NBURN=400 CTYPE=3

            PRINT=10 NOINTER NOHABORT SIGL=8 RANMETHOD=3S3P


$ESTIMATION METHOD=IMP LAPLACE LIKE PRINT=1 NITER=30 AUTO=1 NOINTER

           NOHABORT SIGL=8 RANMETHOD=3S3P MAPITER=0 EONLY=1


$TABLE      ID TIME DV DV2 TRT ONEHEADER NOPRINT

            FILE=sdtab400
```
