# Supplementary material for: Handling underlying discrete variables with bivariate mixed hidden Markov models in NONMEM
Source: J Pharmacokinet Pharmacodyn. 2019 Oct 26;46(6):591–604. doi: 10.1007/s10928-019-09658-z (PMC6868114; doi:10.1007/s10928-019-09658-z)
Supplement: Supplementary file 2 — Supplementary material 2 (PDF 396 kb) [file 10928_2019_9658_MOESM2_ESM.pdf]

Appendix 2

Table A1: Relative root mean squared error (%) of the parameters in the model based on the different evaluated scenarios (see table 2 for details).

Parameters	Base scenario	Scenarios exploring effect of transition probabilities magnitude	Scenarios exploring effect of drug effect magnitude				Scenarios exploring effect of inter individual variability magnitude				Scenarios exploring effect of correlation magnitude		Scenarios exploring trial design	
	1	2	3	4	5	6	7	8	9	10	11	12	13	14
$FEV1_R$	0.799	0.682	0.802	0.776	0.804	0.805	0.722	0.725	0.779	0.759	0.803	0.786	0.792	0.807
$FEV1_E$	1.33	1.44	1.66	1.26	1.3	1.28	1.32	1.28	1.56	1.42	1.49	1.46	2.69	2.69
PRO_R	0.578	0.589	0.611	0.542	0.559	0.514	0.556	0.564	0.534	0.567	0.61	0.536	0.537	0.536
PRO_E	3.48	2.67	3.24	3.17	3.5	2.67	3.34	3.6	3.07	3.12	3.07	3.06	3.7	3.15
$P(S_{t=0} = R)$	1.75	1.86	1.73	1.79	1.76	1.95	1.7	1.52	1.72	1.71	1.77	1.45	1.74	1.88
π_{RE}	7.23	4.81	7.65	8.07	5.23	5.07	4.71	6.1	3.62	4.7	8.05	6.61	8.28	5.98
π_{ER}	3.52	2.47	4.49	3.53	3.16	2.33	3.65	3.92	2.58	2.5	4.15	2.97	4.4	2.74
SLP	10	7.58	6.67	17.6	4.61	12.4	9.61	10.3	7.24	9.3	10.6	8.81	10.8	8.31
σ^2_{FEV1R}	0.967	1.06	0.971	1.14	1.07	1.01	0.721	1.03	0.961	1.08	1.01	1.02	2.26	2.39
σ^2_{FEV1E}	2.28	2.62	2.67	2.11	2.55	2.34	2.31	2.26	2.34	2.9	2.23	2.86	5.63	6.26
σ^2_{PROR}	0.899	1.12	0.9	0.898	1.07	1.14	0.957	1.03	0.905	1.01	0.917	1.03	1.14	1.04
σ^2_{PROE}	2.43	2.39	2.62	2.06	2.84	2.54	2.1	2.37	2.61	2.36	2.24	2.6	2.67	3.02
ρ_R	1.9	2.11	1.94	1.78	2.04	1.88	1.79	1.94	1.85	2.06	0.637	0.598	3.62	4.15
ρ_E	4.65	4.75	4.65	4.68	5.04	4.27	4.77	4.05	4.69	4.72	1.3	1.61	9.82	9.67
ω^2_{FEV1R}	5.77	7.2	5.86	6.39	6.18	5.96	5.98	6.2	6.11	7.18	5.89	6.88	5.87	7.12
ω^2_{FEV1E}	19.5	17.1	20.5	15.2	18.6	14.9	29.7	19.1	30.2	16.3	17.8	20.6	74.7	69.9
ω^2_{PROR}	7.05	6.44	5.81	5.93	6.43	6.28	6.03	6.44	6.03	6.99	5.92	6.74	6.26	6.06
ω^2_{PROE}	7.49	7.27	8.18	7.39	8.36	7.1	8.14	8.22	7.58	6.9	7.66	6.41	7.75	8.28
$\omega^2_{\pi RE}$	187	106	199	161	130	85.5	*NA*	80.9	*NA*	41.8	199	124	187	113
